# Identification of sensory and motor nerve fascicles by immunofluorescence staining after peripheral nerve injury

**DOI:** 10.1186/s12967-021-02871-w

**Published:** 2021-05-13

**Authors:** Xijie Zhou, Jian Du, Liming Qing, Thomas Mee, Xiang Xu, Zhuoran Wang, Cynthia Xu, Xiaofeng Jia

**Affiliations:** 1 Department of Orthopaedics, The Second Affiliated Hospital and Yuying Children′S Hospital of Wenzhou Medical University, Wenzhou, 325027 China; 2 Department of Neurosurgery, University of Maryland School of Medicine, 10 South Pine Street, MSTF Building 823, Baltimore, MD 21201 USA; 3 Department of Orthopaedics, University of Maryland School of Medicine, Baltimore, MD 21201 USA; 4 Department of Anatomy and Neurobiology, University of Maryland School of Medicine, Baltimore, MD 21201 USA; 5 Department of Biomedical Engineering, The Johns Hopkins University School of Medicine, Baltimore, MD 21205 USA; 6 Department of Anesthesiology and Critical Care Medicine, The Johns Hopkins University School of Medicine, Baltimore, MD 21205 USA

**Keywords:** Peripheral nerve, Immunofluorescence staining, Motor fascicles, Sensory fascicles

## Abstract

**Background:**

Inappropriate matching of motor and sensory fibers after nerve repair or nerve grafting can lead to failure of nerve recovery. Identification of motor and sensory fibers is important for the development of new approaches that facilitate neural regeneration and the next generation of nerve signal-controlled neuro-prosthetic limbs with sensory feedback technology. Only a few methods have been reported to differentiate sensory and motor nerve fascicles, and the reliability of these techniques is unknown. Immunofluorescence staining is one of the most commonly used methods to distinguish sensory and motor nerve fibers, however, its accuracy remains unknown.

**Methods:**

In this study, we aim to determine the efficacy of popular immunofluorescence markers for motor and sensory nerve fibers. We harvested the facial (primarily motor fascicles) and sural (primarily sensory fascicles) nerves in rats, and examined the immunofluorescent staining expressions of motor markers (choline acetyltransferase (ChAT), tyrosine kinase (TrkA)), and sensory markers [neurofilament protein 200 kDa (NF-200), calcitonin gene-related peptide (CGRP) and Transient receptor potential vanillic acid subtype 1 (TRPV1)]. Three methods, including the average area percentage, the mean gray value, and the axon count, were used to quantify the positive expression of nerve markers in the immunofluorescence images.

**Results:**

Our results suggest the mean gray value method is the most reliable method. The mean gray value of immunofluorescence in ChAT (63.0 ± 0.76%) and TRKA (47.6 ± 0.43%) on the motor fascicles was significantly higher than that on the sensory fascicles (ChAT: 49.2 ± 0.72%, P < 0.001; and TRKA: 29.1 ± 0.85%, P < 0.001). Additionally, the mean gray values of TRPV1 (51.5 ± 0.83%), NF-200 (61.5 ± 0.62%) and CGRP (37.7 ± 1.22%) on the motor fascicles were significantly lower than that on the sensory fascicles respectively (71.9 ± 2.32%, 69.3 ± 0.46%, and 54.3 ± 1.04%) (P < 0.001). The most accurate cutpoint occurred using CHAT/CRCP ratio, where a value of 0.855 had 100% sensitivity and 100% specificity to identify motor and sensory nerve with an area under the ROC curve of 1.000 (P < 0.001).

**Conclusions:**

A combination of ChAT and CGRP is suggested to distinguish motor and sensory nerve fibers.

## Background

Peripheral nerve injury can lead to the loss of motor, sensory and autonomic nerve function in the body's ganglion segment, which seriously affects patients’ quality of life [1]. Mismatched nerve fascicle repair after peripheral nerve injury may result in partial or complete loss of function [2]. Despite several decades of progress in research and surgical techniques, surgeons still rely on experience to estimate the characteristics of damaged motor or sensory nerve stumps and perform a differentiated fascicular repair. Thus, a satisfactory recovery is often difficult to achieve [3]. We have shown a 3-dimensional, printed scaffold repair technology promoting neural regeneration with bifurcating sensory and motor pathways after complex peripheral nerve injuries [4]. Therefore, the ability to identify and differentiate motor and sensory fascicles is greatly beneficial to the development of new approaches to facilitate neural regeneration. In addition, the ability to identify motor and sensory fascicles is crucial to the development of next generation nerve signal-controlled neuro-prosthetic limbs with sensory feedback technology, which is connected to residual peripheral nerves through the neural interface via intrafascicular electrodes as we reported [5–7]. It’s particularly important to reliably distinguish motor and sensory nerve fascicles to properly transmit signals via microelectrode as motor order or sensory feedback via the regenerated nerves [7].

Currently, there are four reported methods to distinguish sensory and motor nerve fascicles: anatomical, electrophysiological, infrared spectrum, and enzymohistochemical staining [8–11]. The anatomical method has been widely used in the past decades, but surgeons can only rely on their own experience to estimate the type of nerve fascicle, and may cause partial or total loss of nerve function after nerve transplantation [12]. Therefore, the anatomical technique alone is difficult to effectively gauge the type of fascicle. Electrophysiological methods such as evoked potentials were used to distinguish sensory from motor nerve fibers. While we have extensive experience with the electrophysiological study of nerve injury [5, 6, 13, 14], this method can only distinguish the main nerve branch; it is unable to show the composition of the nerve fiber. Moreover, there is a need for larger sample sizes to study the electrophysical technique due to the high variance of measurements seen in current studies [15]. Infrared spectrum identification requires a variety of equipment, complex calculations, and many interference factors, which limits its application to a great extent [10]. Enzymohistochemistry staining, including immunofluorescence staining and immunohistochemical staining, is currently one of the most frequently used methods. However, it is difficult to distinguish the results using immunohistochemistry after labeling with a multi substrate color system. In addition, the color of the substrate and the thickness of the slice will affect the final result, and the chromogenic substrate is an enzymatic reaction that easily saturates the substrate, thus limiting the semi quantitative analysis [16]. Compared with immunohistochemistry, immunofluorescence can carry out a reaction with multiple markers. Antibody-coupled fluorescence can also increase the resolution [16].

Although immunofluorescence is widely used, the efficacy/accuracy of popular immunofluorescence markers for motor and sensory nerve fibers remains unclear. In addition, the results of quantitative processing methods for fluorescent images are different without a proper comparison. Although almost all peripheral nerves are a mixture of motor and sensory nerves, the facial nerve is mainly composed of motor nerve fibers and the sural nerve is mainly composed of sensory nerve fibers [17, 18]. We hypothesize that preferable markers will be evaluated and/or an optimal combination will be selected to identify and differentiate the motor and sensory axons using immunofluorescence staining in this side by side comparison in the facial and sural nerve. In this study, we selected the relatively pure facial and sural nerves as either the motor or sensory fascicles for staining, and examined the expressions of available and commonly used motor markers [choline acetyltransferase (ChAT), tyrosine kinase receptor A (TrkA)] and sensory markers [neurofilament protein 200 kDa (NF-200), calcitonin gene-related peptide (CGRP) and Transient receptor potential vanillic acid subtype1 (TRPV1)] using immunofluorescence staining aiming to evaluate the best differentiative approach. These markers have been widely used to label motor or sensory nerve fascicles experimentally [19, 20], however their quantified preferential staining in peripheral nerves after peripheral nerve injury have not been studied.

## Materials and methods

### Experimental procedures

From 5 fresh euthanized nude rats, weighing 200 g to 250 g, we obtained healthy facial nerve and sural nerve specimens. To expose the facial nerve trunk and its branches, a 1 cm skin incision on a horizontal plane was performed from the inferior margin of the auricular, extending anteriorly. Facial nerve specimens were obtained by transecting at the stylomastoid foramen and 5 mm distal to the skin incision, thus extracting a 10 mm nerve segment. A 1-cm long sural nerve segment was dissected from the sciatic nerve. We obtained both sides (10 facial nerve specimens and 10 sural nerve specimens) in all rats, and randomly selected one side of each nerve type for the following studies. Differentially expressed proteins could be observed in proximal and distal nerve segments due to different cells and extracellular matrix of proximal and distal nerve segments [21]. In our study, all the nerve sections were collected from the similar position of nerves. Experimental protocols were approved by the IACUC of University of Maryland School of Medicine Animal Care and Use Committee.

The nerve specimens were immersion-fixed in the 4% Paraformaldehyde (PFA) fixative for 24 h and then placed into 30% sucrose 0.1-M phosphate-buffered saline (PBS) for at least 48 h. The sural and facial nerves were frozen, and then 10 µm thick transverse serial sections were obtained using a freezing microtome (Leica, Germany) at − 20 °C. All sections were stored at − 20 °C. Three slides were randomly selected from each specimen for quantitative immunofluorescence staining in five samples total.

### Immunofluorescence analysis

Standard Immunofluorescence procedures were followed [22]. The facial nerve and sural nerve sections were incubated with the primary antibody against ChAT (rabbit, diluted 1: 100; Millipore), NF-200 (rabbit, diluted 1: 200; Sigma), CGRP (mouse, diluted 1: 200; Abcam), TRKA (rabbit, diluted 1: 200; Abcam), TRPV1 (mouse, diluted 1: 200; Abcam) followed by 24 h at 4 °C. Following incubation with the primary antibody, the specimens were then washed again three times (5 min/wash) in PBS and incubated in the secondary antiserum solution. Antigens of facial nerve sections were observed by using Invitrogen Alexa Fluor-594 donkey anti-rabbit secondary antibody, Invitrogen Alexa Fluor 488-conjugated goat anti-rabbit and Abcam Alexa Fluor 488-conjugated goat anti-mouse (diluted 1: 500; USA) 2 h at 37 °C. Autofluorescence (negative control) that incubated with secondary antibody only were used during each staining.

After being washed, all sections were covered and mounted with ProLong™ Gold Antifade Mountant with DAPI (4′,6-diamino-2-phenylindole, blue, Invitrogen, USA). Micrographs were obtained by using a Leica DMi8 microscope camera equipment (Leica Microsystems). All fluorescent images were analyzed in Image J (National Institutes of Health, USA), as we previously described [23, 24]. Each data point is from five experimental animals. Three tissue sections from selected nerves were collected from each animal in a blinded fashion. Quantification was conducted by counting the axons with immunoreactivity in 3 randomized microscopic fields in each section. For the immunofluorescence analysis, area percentage and mean gray value were analyzed using Image J software (v1.8.0, NIH, USA). After converting the image to black and white with 8-bit type, the threshold of the image was adjusted to best cover the axon area (Image-Adjust-Threshold-Apply); then the area of the axon was measured and recorded (Analyze-Measure) after randomly selecting three fixed areas (50 μm * 50 μm) on each image. The area percentage, mean gray value, and axon count were automatically calculated using image J.

### Statistics

An independent t-test was employed to determine statistical differences of quantified immunofluorescence area and mean gray value between two groups using SPSS version 22.0 (IBM Corp., Armonk, NY, USA). Image J software was used to determine the immunofluorescence area and mean gray value (Version 1.8.0). A receiver operating characteristic (ROC) curve was constructed to determine the cut-points of the mean gray value of ChAT and CGRP and the ChAT/CGRP ratio, with optimal sensitivity and specificity to identify motor and sensory fascicles. All values were expressed as a mean ± SEM. Statistical significance was set at P < 0.05.

## Results

### The expressions of motor markers in motor and sensory fascicles

**Fig. 1:**
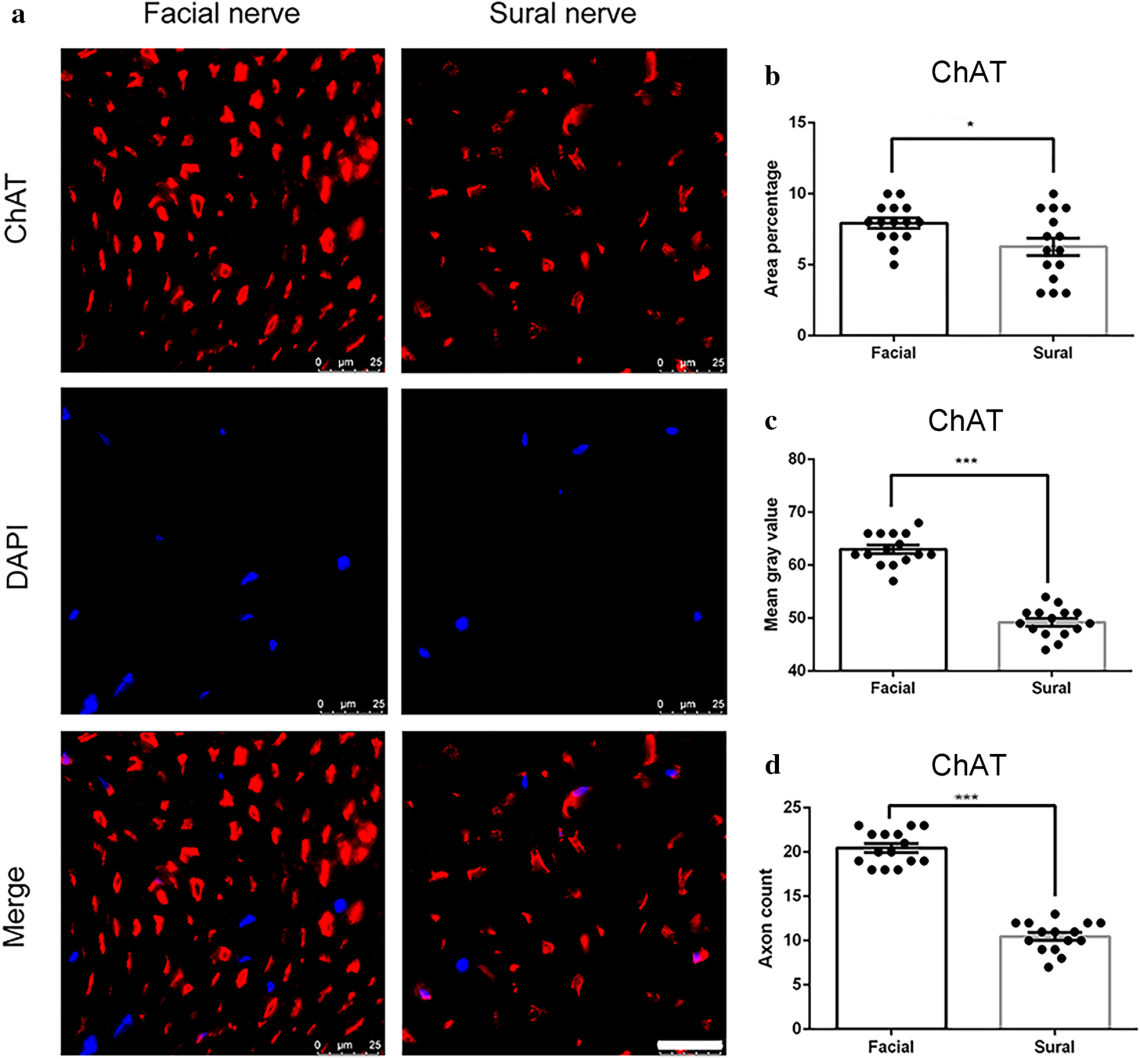
ChAT immunofluorescence staining of the facial nerve and sural nerve. **a** representative image of staining with ChAT (red) and DAPI (blue). Scale bar is 25 μm. Quantification of **b** area percentage; **c** mean gray value; and **d** axon count of positive ChAT staining

ChAT is an enzyme synthesized within motor axons, and ChAT immunofluorescence is dominantly expressed in motor fascicles [25]. Our study showed that the area percentage of ChAT-labeled axons (red staining) in the facial nerve (7.9 ± 0.36%, Fig. 1a) was significantly higher than the sural nerve (6.3 ± 0.62%, Fig. 1b, P < 0.001). The mean gray value and axon count of ChAT was also significantly higher in the facial nerve (63.0 ± 0.76%, 20.47 ± 0.4%), compared to the sural nerve (49.2 ± 0.72%, 10.47 ± 0.4, P < 0.001) (Fig. 1c, d).

**Fig. 2:**
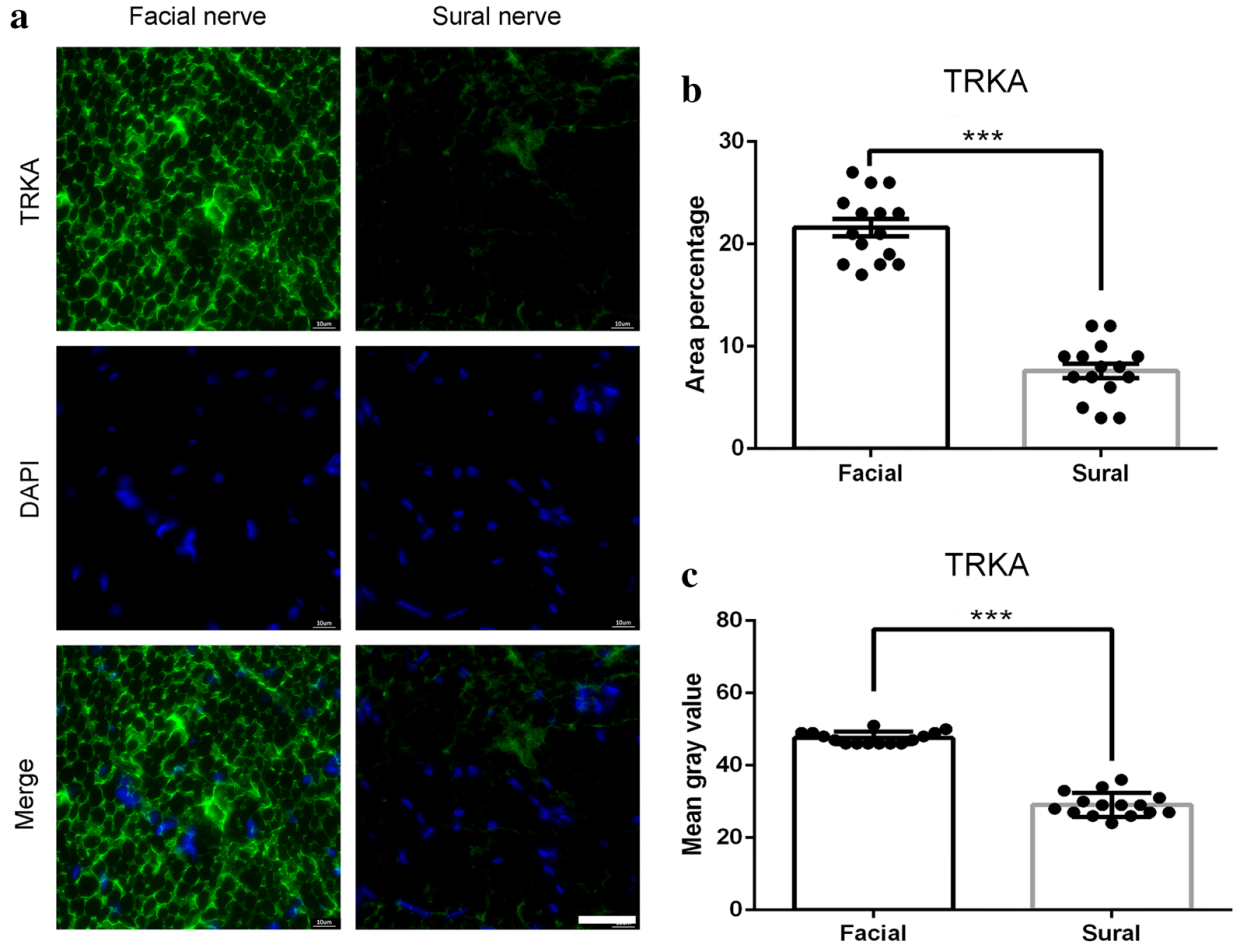
TRKA immunofluorescence staining of the facial nerve and sural nerve. **a** representative image of staining with TRKA (green) and DAPI (blue). Scale bar is 25 μm. Quantification of **b** area percentage and **c** mean gray value

TrkA was reported to detect the function of extraocular motoneurons and spinal motor neurons after axotomy or in neurodegenerative diseases [26]. Our results showed that TrkA is mainly expressed on the cell membrane and strongly expressed in the facial nerve (Fig. 2a). The average immunofluorescence area was 21.6 ± 0.84%, significantly higher than that in sural nerve (7.6 ± 0.72%, P < 0.001) (Fig. 2b). The results were also confirmed with mean gray values of TrkA (47.6 ± 0.43% vs. 29.1 ± 0.85%, P < 0.001, Fig. 2c) in the facial nerve and sural nerve.

### The expressions of sensory markers in motor and sensory fascicles

**Fig. 3:**
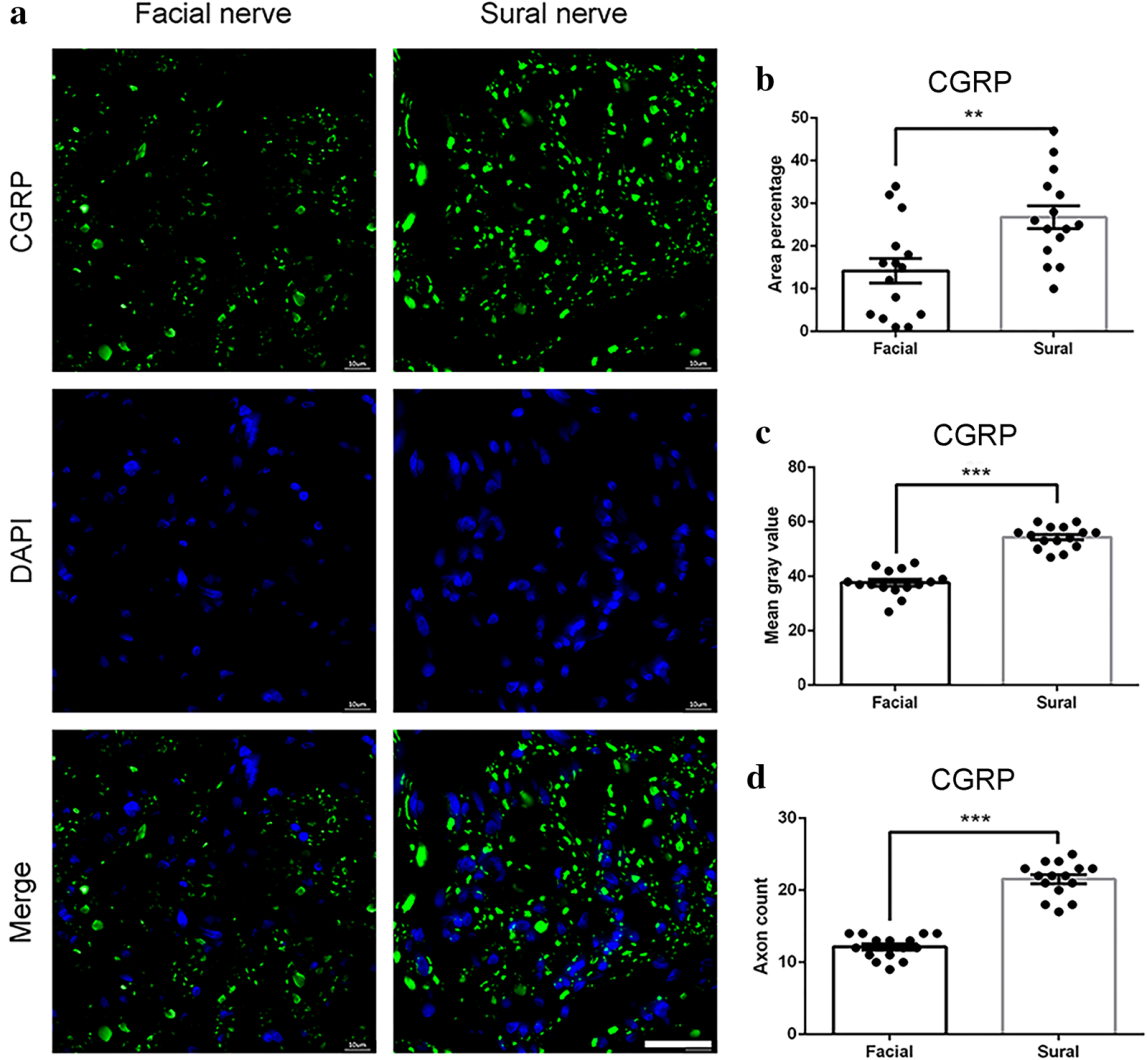
CGRP immunofluorescence staining of the facial nerve and sural nerve. **a** representative image of staining with CGRP (green) and DAPI (blue). Scale bar is 25 μm. Quantification of **b** area percentage; **c** mean gray value; and **d** axon count of positive CGRP staining

CGRP is mainly expressed in primary afferent neurons and plays an important role in the repair of axonal injury [27]. From Fig. 3a we observed more positive expression of CGRP in the sural nerve than in the facial nerve. The area percentage was nearly 2 times higher when comparing the fluorescence staining in the sural nerve with the facial nerve (26.7 ± 2.68% vs. 14.2 ± 2.85%, P < 0.001, Fig. 3b). The mean gray value also indicated higher CGRP expression in the sural nerve (54.3 ± 1.04%) than in facial nerve (37.7 ± 1.22%, P < 0.001, Fig. 3c). The axon count was significantly higher in sural nerve compared with that of the facial nerve (21.53 ± 0.6 vs. 12.13 ± 0.4, P < 0.001, Fig. 3d).

**Fig. 4:**
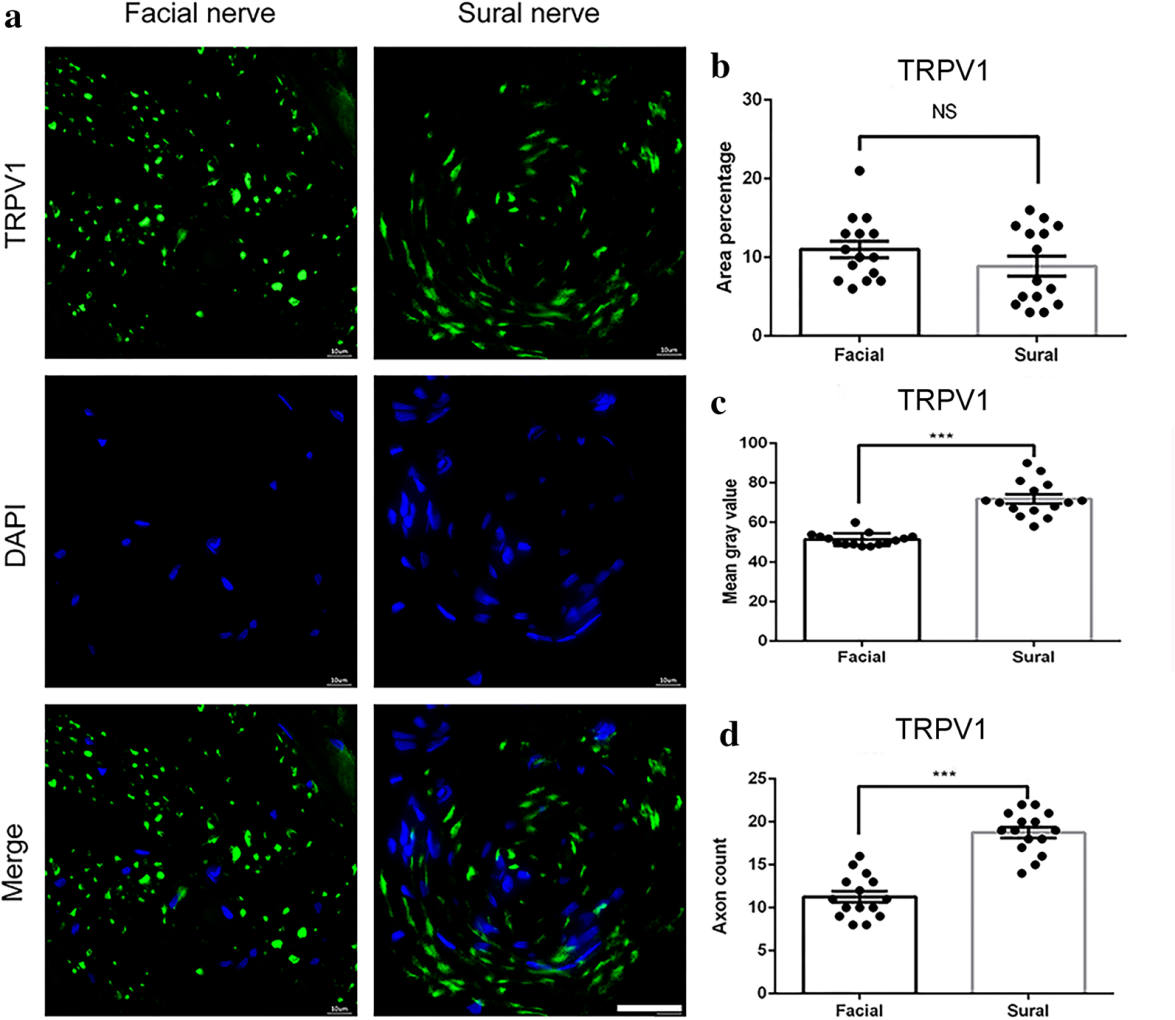
TRPV1 immunofluorescence staining of the facial nerve and sural nerve. **a** representative image of staining with TRPV1 (green) and DAPI (blue). Scale bar is 25 μm. Quantification of **b** area percentage; **c** mean gray value; and **d** axon count of positive TRPV1 staining

TRPV1 is sensitized in the process of inflammation and injury and expressed in peptidergic and nonpeptidergic nociceptors [28, 29]. We found positive expression of TRPV1in both the sural and the facial nerve fibers (Fig. 4a). There was no significant difference in the average area percentage between the two groups (11.0 ± 1.05% vs. 8.8 ± 1.26%, P > 0.05, Fig. 4b). Both TRPV1 mean gray values (51.5 ± 0.83% and 71.9 ± 2.32%, P < 0.001, Fig. 4c) and axon count (11.27 ± 0.6 and 18.73 ± 0.6 for the facial nerve and sural nerve, P < 0.001, Fig. 4d) were significantly higher in the sural nerve than in the facial nerve fibers.

**Fig. 5:**
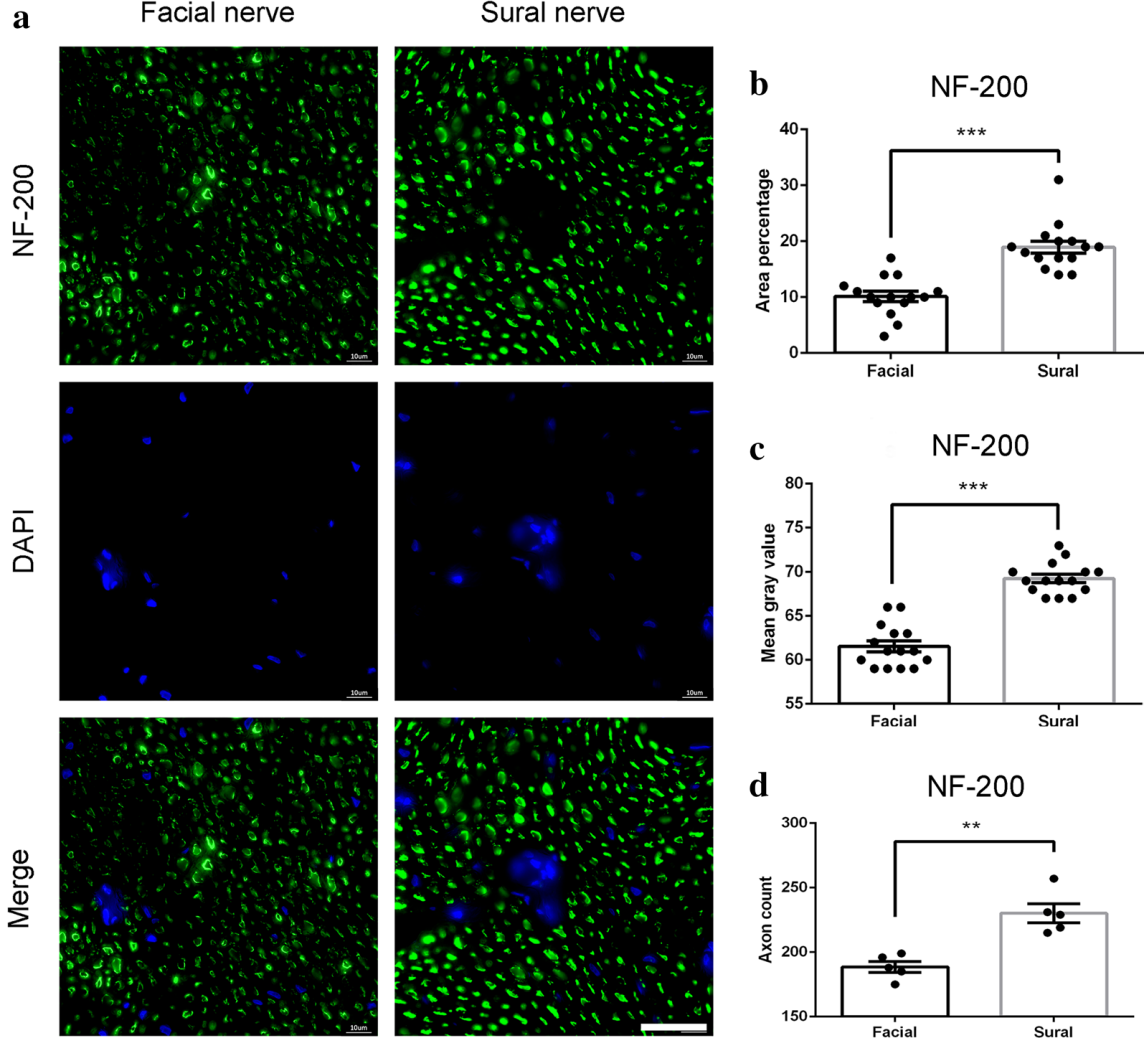
NF-200 immunofluorescence staining of the facial nerve and sural nerve. **a** representative image of staining with NF-200 (green) and DAPI (blue). Scale bar is 25 μm. Quantification of **b** area percentage; **c** mean gray value; and **d** axon count of positive NF-200 staining

NF-200 is an axon-specific intermediate filament found in peripheral nerves and is used as a marker for sensory myelinated fibers in previous studies [30, 31]. Our results showed NF-200 was positive in both sural and facial nerve fibers (Fig. 5a). The area percentage results showed that NF-200 had a significantly higher positive rate in sural nerve fibers (18.9 ± 1.08%) compared to facial nerve fibers. NF 200 was expressed in facial nerve fibers with the rate of 10.1 ± 0.90% (Fig. 5b). Furthermore, the mean gray value analysis showed the rate is 69.3 ± 0.46% in the sural nerve and 61.5 ± 0.62% in the facial nerve (Fig. 5c). Similarly, a higher stained axon count was observed in the sural nerve (30.20 ± 0.7) than that observed in the facial nerve (21.53 ± 0.5, P < 0.001) (Fig. 5d).

### The optimal combination of target markers to differentiate motor and sensory fascicles

**Fig. 6:**
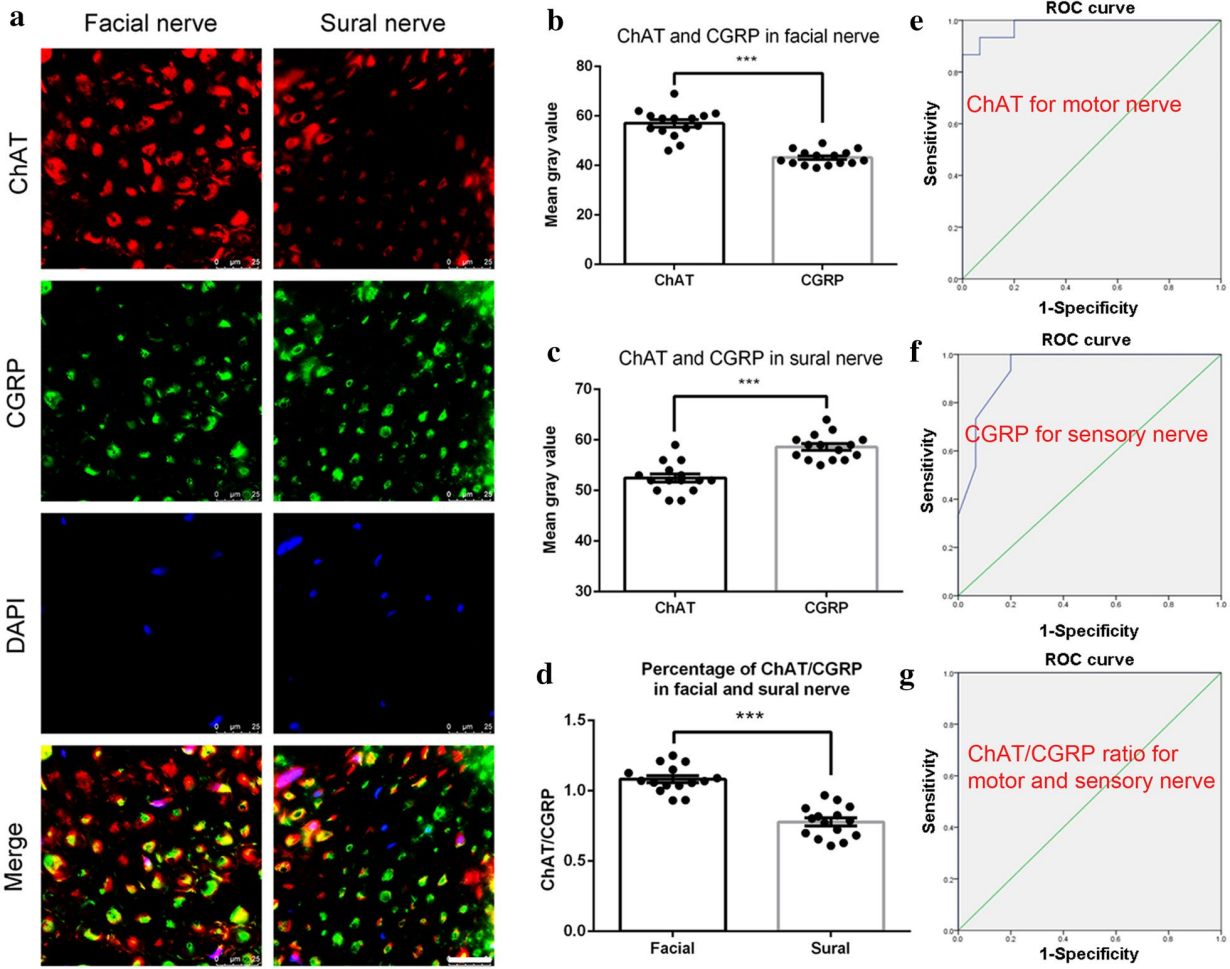
Double immunofluorescence staining results of ChAT and CGRP in facial and sural nerve. **a** representative image of staining with ChAT (red), CGRP (green), and DAPI (blue). Scale bar is 25 μm. Quantification of **b** area percentage; **c** mean gray value; and **d** axon count of positive ChAT/CGRP staining. Mean gray value cut-points of **e** CHAT to identify motor nerve fascicles and **f** CGRP to identify sensory nerve fascicles were determined using ROC curve methodology with sensitivity and specificity. **g** The most accurate cut-point occurred using CHAT/CRCP: a cutpoint of 0.855 yielded 100% sensitivity and 100% specificity identifying motor and sensory nerve fascicles with an area under the ROC curve of 1.000 (P < 0.001)

Based on the above results, we selected ChAT and CGRP as the optimal combination of target markers to distinguish motor and sensory nerve fibers and performed double immunofluorescence staining (Fig. 6a). The quantified mean gray value based on the positive ChAT in facial nerve was 57.0 ± 1.48%, which was significantly higher than CGRP in facial nerve 52.4 ± 0.76% (P < 0.001) (Fig. 6b). Furthermore, the mean gray value of CGRP in the sural nerve (43.1 ± 0.77%) was significantly higher than that of ChAT in the sural nerve (58.6 ± 0.65%, P < 0.001) (Fig. 6c). There is a significant difference (P < 0.001) by the mean gray value comparison between ChAT and CGRP, which showed the ratio of 1.1 ± 0.03% and 0.7 ± 0.02% for the facial nerve and sural nerve, respectively (Fig. 6d).

Accurate cut-points of the mean gray values were determined using ROC curve methodology. A cut-point of 50.5 for CHAT had 86.7% sensitivity and 100% specificity identifying motor nerve fascicles with an area under the ROC curve of 0.982 (P < 0.001) (Fig. 6e), and a cut-point of 54.5 for CGRP had 100% sensitivity and 80% specificity identifying sensory nerve fascicles with an area under the ROC curve of 0.940 (P < 0.001) (Fig. 6f). The most accurate cut-point occurred using CHAT/CRCP ratio, where a value of 0.855 had 100% sensitivity and 100% specificity to identify motor and sensory nerve with an area under the ROC curve of 1.000 (P < 0.001) (Fig. 6g). All results indicated that ChAT was highly expressed in motor facial nerve fibers, while CGRP is more dominant in sensory sural nerve fibers, and a combination of ChAT and CGRP is an optimal combination to distinguish motor and sensory nerve fibers.

## Discussion

Identification of motor and sensory fibers is important not only to develop new approaches to facilitate neural regeneration, but also crucial to develop the future generation of nerve signal-controlled neuro-prosthetic limbs with close-loop sensory feedback. The use of neuroprostheses to fully replace amputated limbs or recover their function after injury is still challenging [32, 33]. To achieve a natural control of the limb movement, the selective function of motor and sensory nerves and their fascicles should be properly selected for the neuroprosthesis [34]. Such selectivity is an unsolved challenge. Nerve mapping in amputees or in peripheral nerve injuries is not possible using intraoperative neuromonitoring techniques [35]. Thus, the surgeon must exclusively rely on the nerve topography for the implantation. Here our results showed the simple and useful immunofluorescent approach to distinguish motor and sensory nerves, by which micro-suture marked nerve stumps could be identified and prepared for interfacing at second stage. It is a simple tool to identify the motor and sensory nerves after trauma and provides useful information for the next step of the operation. Thus, it can be a complementary approach, which might help in the planning of the interfacing procedure and move the field one step closer to a comprehensive solution for the application of neuro-prosthetic limbs in a clinical setting.

We selected five commonly used biomarkers that have been used for identification of either motor or sensory axons and evaluated immunofluorescent staining in side-by-side comparisons, controlling for time and other conditions. Our study shows that nerve fibers can be distinguished effectively by using ChAT for motor nerve fibers and CGRP for sensory nerve fibers, and ChAT and CGRP were the optimal combination of markers for motor and sensory fascicles, respectively. We used three methods, including the average area percentage, the mean gray value, and the axon count, to quantify the positive expression of nerve markers in the immunofluorescence images, and showed the mean gray value method is a more stable method. Since other, available solutions are scarce, this method will provide a convenient, fast, and reliable methodology to distinguish primarily motor fascicles from primarily sensory fascicles after peripheral nerve injury and will have a great translational value in the clinic. This technique enables a differentiated fascicle repair that will greatly improve the success rate of nerve repair and functional recovery after surgery. It will also be a very important addition for future neural prostheses, which makes neural motor signal control and sensory signal feedback technology feasible.

We have screened nearly all popular immunofluorescence markers in the literature and examined a panel of the five most effective ones including motor markers (ChAT and TRKA) and sensory markers (CGRP, TRPV1, and NF-200) in the sural nerve (primarily sensory nerve fibers) and the facial nerve (primarily motor nerve fibers). Although these markers have been suggested to be preferably used in either motor or sensory axons, the side-by-side comparison has never been assessed before. Since there is no available tool to identify the fascicles correctly as motor or sensory, the sensitivity and specificity of these markers in motor or sensory fascicles cannot be calculated. Thus, in our study we define it as efficacy of preferential staining, which refers to whether the antibody can show a positive result in the desired tissue, and how much the expression of the positive result is the antibody. In this respect, all the antibodies we selected were expressed to varying degrees on both nerve fibers, but not specifically enough. With the strong positive antibodies that were expressed, the maximum positive area was about 30% when the average area percentage was used for data analysis. The axon count is up to 10–30% of the positively stained area in the whole selected microscopic fields, which is still high, as the space outside the axon contains a large amount of myelin sheath that cannot be shown in immunofluorescence without properly specialized antibodies. Using mean gray value, the positive rate reaches 60–80%, thus the mean gray value methodology has a higher efficacy of preferential staining and is a more stable method.

To select the best analysis methods to distinguish between the motor and sensory fibers of injured nerves, we evaluated the efficacy through different analysis methods including fluorescence area percentage, mean gray value, and axon count, which have never been systemically assessed in peripheral nerves yet. When using the average area percentage, the image J software randomly selects areas and calculates highly expressed axons, but ignores low grayscale and weakly expressed nerve fibers. The results show that the percentage obtained by the average area percentage is significantly lower than the mean gray value. Compared with the abovementioned methods, the axons with weak fluorescence can also be detected when the mean gray value is applied. Using axon counting is a generally accepted method, which can be popularly used in the slides of 100× under an optical microscope. However, it should be noted that when using a 40× magnified image, an axon with a smaller diameter may not be found. That reduces the resolution of some axons, making the accuracy of all axon counting unstable [36]. In addition, the method is based on the number of circular axons, which ignores compressed or oblique sub-areas in the nerve tissue sections that can easily lead to inaccurate axon count [37]. Therefore, we recommend using the mean gray value to quantitatively detect the immunofluorescence results, because of its high efficacy of preferential staining and relative stability.

ChAT, as a primary motor fascicles marker, is widely reported to be highly expressed in human facial motor neurons and rat facial motor neurons [38, 39], with high concentrations in motor neurons in both the central nervous system (CNS) and peripheral nerve axons [40, 41]. A key feature of motor neuron development and function is the expression of the acetylcholine biosynthetic enzyme, ChAT [42, 43]. Yuan et al. used ChAT as an enzymatic marker in immunohistochemistry staining to detect motor axons and used NF200 to detect sensory axons and showed that the sciatic nerve contains both motor and sensory axons [44]. Castaneda and Wang et al. showed the same in their studies [45, 46]. However, these studies only show that ChAT and NF200 can be expressed positively in motor nerve fibers and sensory nerve fibers, without quantitative elaboration on the accuracy and efficacy of preferential staining of this method. The other marker, TrKA is mainly expressed on the cell membrane and strongly in the facial nerve in our study. Carrizosa et al. chose TrkA positive expression to detect the function of spinal motor neurons and extraocular motoneurons [26]. In the CNS, within the striatal motor neurons and the septal/diagonal band complex, TrkA neurons (> 99 and > 95%, respectively) co-expressed ChAT [47]. Recently, Han et. al showed that TrkA was expressed in primary sensory fibers mainly concentrated in Dorsal root ganglion (DRG) [48]. TrkA’s expression in peripheral sensory nerve fascicles is unknown. Our study showed TrkA is not obviously expressed in the sural nerve. ChAT is a protease that is scattered in the cells and the different branches of the facial nerve may have variable compositions. Therefore, although our results show that the average overall density is not very high, it is reasonable and sufficient to distinguish between two different types of nerve fibers by using this method. Thus, comparing all other markers evaluated, we recommend using ChAT to detect motor nerve fibers because they have the highest efficacy of preferential staining and accuracy.

NF-200 was used as a marker for sensory myelinated fibers and the regeneration of sensory nerve axons [30]. However, NF-200, an intermediate filament protein, is a cytoskeletal structure that can be found in any mature axon [49]. NF200 was not only expressed in the majority of myelinated DRG neurons and peripheral nerves [50], but also is used to identify large sensory neurofilaments [29, 51]. TRPV1 is widely distributed in nociceptors and receives external stimulus signals [52]. It covers a large spectrum of pain qualities, from chemical to thermal, as a peripheral pain-modulating target [53]. This ion channel that is abundantly expressed in the peripheral sensory system [53]. It is worth noting that the concentration of NGF in surrounding tissues might affect the activity of TRPV1 and up-regulate its expression [54]. TRPV1 is a mechanosensitive receptor that is commonly found throughout sensory C fibers; when activated, it releases the neurotransmitter CGRP [55]. Typically used as a marker for peptidergic sensory nerves, CGRP plays a pivotal role in trigeminal system, pain and temperature sensation [56]. Furthermore, it is expressed in other sensory neurons as a marker of neurons essential for heat responses in peripheral nerves [57, 58]. CGRP is preferably expressed on sensory nerve fibers, and its role is to maintain the release of neurotrophic factors, activation of protein kinases, opening of cation channels, and amplify pain signals [55]. CGRP may be expressed more during nerve regeneration after peripheral nerve injury, however it has not been studied due to lack of proper control. Our study showed for the first time that CGRP is more reliable to distinguish the sensory nerve fascicles from the motor nerve fascicles.

Although the activity of the ChAT enzyme is affected by different tissues and time points, it has been reported that the activity of ChAT is highest following axotomy and gradually declines over time [59]. We experimented with ChAT within 1 day after nerve harvest, in hopes of maximally retaining the enzymatic activity. Sensory neurons are considerably more “plastic” with respect to specification than motor neurons [60]. Studies suggest that sensory neurons are intrinsically specified with respect to their peripheral targets [61] and subclasses of sensory neurons show different integrin expression [60]. Thus, we chose three different sensory markers for comparison and selected the best one. While these markers may be expressed differently in CNS or other organs, this study examines only its efficacy of preferential staining after peripheral nerve injury. Overall, this work offers a meaningful view of the development of immunostaining which satisfies the criteria of rapidity, simplicity, cost-effectiveness, high efficacy of preferential staining and reproducibility for the identification of nerve fascicles.

## Conclusion

In this study, immunofluorescence staining was used to identify motor nerve fibers and sensory nerve fibers with five popular markers, and to evaluate their efficacy by different analysis methods. It is suggested that ChAT and CGRP are the optimal combination to differentiate the motor and sensory nerve fascicles by using the mean gray value method. This technique provides a more convenient and reliable method to enable a differentiated nerve fascicle repair that will greatly improve the functional recovery after nerve repair and promote neural regeneration. It helps determine the effectiveness of nerve regeneration achieved by, for example, motor or sensory path specific growth factor guided regeneration and motor or sensory path specific rehabilitation using electrical stimulation. It will also benefit the development of future neural signal-controlled prosthesis with sensory signal feedback technology.

## Data Availability

Not applicable.

## Abbreviations

ChAT: Choline acetyltransferase
CGRP: Calcitonin gene-related peptide
CNS: Central nervous system
DAPI: 4′,6-Diamino-2-phenylindole
DRG: Dorsal root ganglion
NF-200: Neurofilament protein 200 kDa
PBS: Phosphate-buffered saline
TrkA: Tyrosine kinase receptor A
TRPV1: Transient receptor potential vanillic acid subtype1
